# Light therapy with boxes or glasses to counteract effects of acute sleep deprivation

**DOI:** 10.1038/s41598-019-54311-x

**Published:** 2019-12-02

**Authors:** Henri Comtet, Pierre A. Geoffroy, Mio Kobayashi Frisk, Jeffrey Hubbard, Ludivine Robin-Choteau, Laurent Calvel, Laurence Hugueny, Antoine U. Viola, Patrice Bourgin

**Affiliations:** 1 Sleep Disorders Center & CIRCSom (International Research Center for ChronoSomnology), University Hospital, 1 place de l’Hôpital, 67000 Strasbourg, France; 2 CNRS UPR 3212, Institute for Cellular and Integrative Neurosciences, 8 rue du Général Rouvillois, 67000 Strasbourg, France; 3 Paris Diderot University - Paris VII, 5 Rue Thomas Mann, 75013 Paris, France; 4 CEED (European Center for Diabetes Studies), Boulevard Leriche, 67200 Strasbourg, France; 5 Fédération de Médecine Translationnelle de Strasbourg (FMTS), Strasbourg University, 4 rue Kirschleger, 67085 Strasbourg, France

**Keywords:** Sleep deprivation, Wakefulness, Attention, Neurophysiology, Sleep disorders

## Abstract

Sleep deprivation, in the context of shift work, is an increasing major public health issue. We aimed to determine whether early light administration can counteract sleep deprivation effects, and to compare LED-glasses with a traditional light therapy box. This cross-over design study included 18 individuals exposed to light therapy for 30 minutes at 5 am after one night of complete sleep deprivation, to mimic the night shift condition. Individuals were randomly exposed to 10,000 Lux light box, 2,000 Lux LED blue-enriched glasses, and control (ambient dim-light at 8 lux). Alertness, cognition and mood were assessed throughout the night and following morning. Five women and 13 men (mean 24.78 year old) presented with a progressive and increasing alteration of alertness, cognition, and mood during each sleep deprivation. A rebound was observed at 8 am resulting from the circadian drive overriding cumulative sleep homeostatic effects. Morning light significantly improved sleepiness and sustained attention from 5 to 7 am. These effects were comparable between devices and significantly different from control. Both devices were overall well and similarly tolerated. Early morning light therapy in the condition of sleep loss may have broad practical applications to improve sleepiness, sustained attention and subsequent risk of accidents.

## Introduction

It has become increasingly clear that sleep loss affects important brain functions such as alertness, information processing, cognitive function and mood^1–5^. Thus, sleep deprivation, in the context of shift work, is an increasing major public health issue^6–8^. Indeed, it is demonstrated that the effect of shift work on sleep mainly concerns acute sleep loss in connection with night shifts and early morning shifts^9^. Sleep deprivation is associated with increased attention-deficit or somnolence-related accidents, especially during the early morning period, which corresponds to both the nadir of the circadian rhythm of alertness and the homeostatic sleep pressure rise. Incidentally, early morning is the most at-risk period of work-related accidents or traffic accidents^10^. Shift work has also been associated with type 2 diabetes, weight gain, coronary heart disease, stroke, and cancer^9^, leading to great social, economic and human costs including accidents, which points to the urgent need for practical strategies aimed at counteracting sleep deprivation effects.

Light is not only important for vision, but also has strong and pervasive effects on physiology and behavior, including vigilance states in both a circadian and a direct non-circadian manner, as well as by influencing the sleep homeostat^11,12^. Light therapy (LT) is well-established for disorders such as circadian rhythm sleep disorders including shift work, sleep phase delayed disorders, and mood disorders such as seasonal affective disorder and major depressive episodes from unipolar or bipolar disorders^13–15^. Efficacy and tolerance of LT have been well documented, but one potential shortcoming in daily life might be its use for 30 to 60 minutes in the morning. This might be difficult for some patients to be unoccupied during LT. Thus, the development of portable devices such as LT glasses may represent an alternative allowing more freedom to the user and consequently broader clinical applications^16^.

In this context, this work has two objectives. First, we examine if morning LT can enhance vigilance, cognition, and mood; which are all altered with sleep deprivation and are known risk factors for accidents. For this, we tested a 30-minute bright light intervention at 5am, which is close to the end of many conventional night shifts and corresponds to the alertness nadir^17^. Secondly, we aimed to compare the efficacy and tolerance of LT glasses to a traditional LT box.

## Results

### Participants

20 subjects (7 women, 13 men) were recruited, but one woman was excluded because of a depressive episode as assessed by the Beck’s Depression Inventory, and another woman did not respond to our queries during the follow-up and never show up again for unknown reasons. Thus, the study population consisted of 18 subjects (5 women, 13 men), with a mean age of 24.78 years. See Table 1 for detailed characteristics.

**Table 1:** Characteristics of the population (n = 18).

Age (years)	24.78 ± 0.87
Gender (F)	5 (27.78)
BMI (kg/m²)	24.00 ± 1.18
Short Form (36) Survey Score– physical (/100)	90.13 ± 1.40
Short Form (36) Survey Score– mental (/100)	79.59 ± 2.39
Usual Bedtime (hh:mm)	23:45 ± 0:13
Usual Time Out of Bed (hh:mm)	7:19 ± 0:12
Mid Sleep Time (hh:mm)	3:39 ± 0:11
Horne & Ostberg Morningness-Eveningness Score (/86)	45.06 ± 2.39
Pittsburgh Sleep Quality Index Score (/21)	3.67 ± 0.65
Insomnia Severity Index Score (/28)	5.33 ± 0.87
Beck Depression Inventory-II Score (/39)	2.50 ± 0.47
Epworth Sleepiness Scale (/24)	5.86 ± 0.65
Positive and Negative Affect Schedule - positive affect (/50)	32 ± 1.46
Positive and Negative Affect Schedule - negative affect (/50)	13 ± 0.66

Legend: Values are expressed in mean ± SEM., or in N (%).

### Sleepiness

**Figure 1:**
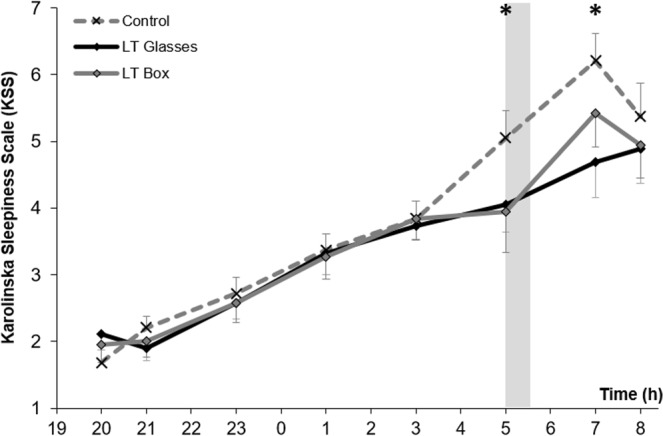
Sleepiness assessment by Karolinska Sleepiness Scale (KSS) between different lighting conditions. Horizontal axis is the time (hours) of the sleep deprived night; vertical axis is the KSS mean score. KSS is ranged from 1 “very alert” to 9 “very sleepy, great efforts to keep awake”. For each group, marks are the mean, bars are the SEM. Bright light therapy is administered at 5am for 30 minutes (gray rectangle).*Control vs. LT < 0.05.

Sleepiness, measured by KSS, increased throughout the night until 3:00, with no significant difference between groups. Sleepiness significantly improved at 5:00 and 7:00 in both LT conditions compared to the control (p < 0.05), an effect which disappeared at 8:00, with no differences between LT box and glasses (Fig. 1). Cohen’s d was used to assess size effect. Compared with controls, the effect size, at 5:00, was 0,52 and 0,58 (medium effect size) respectively for boxes and glasses, whereas, at 7:00, it was 0.42 for boxes (small effect size) and 0,78 for glasses (medium to large effect size).

### Cognition

**Figure 2:**
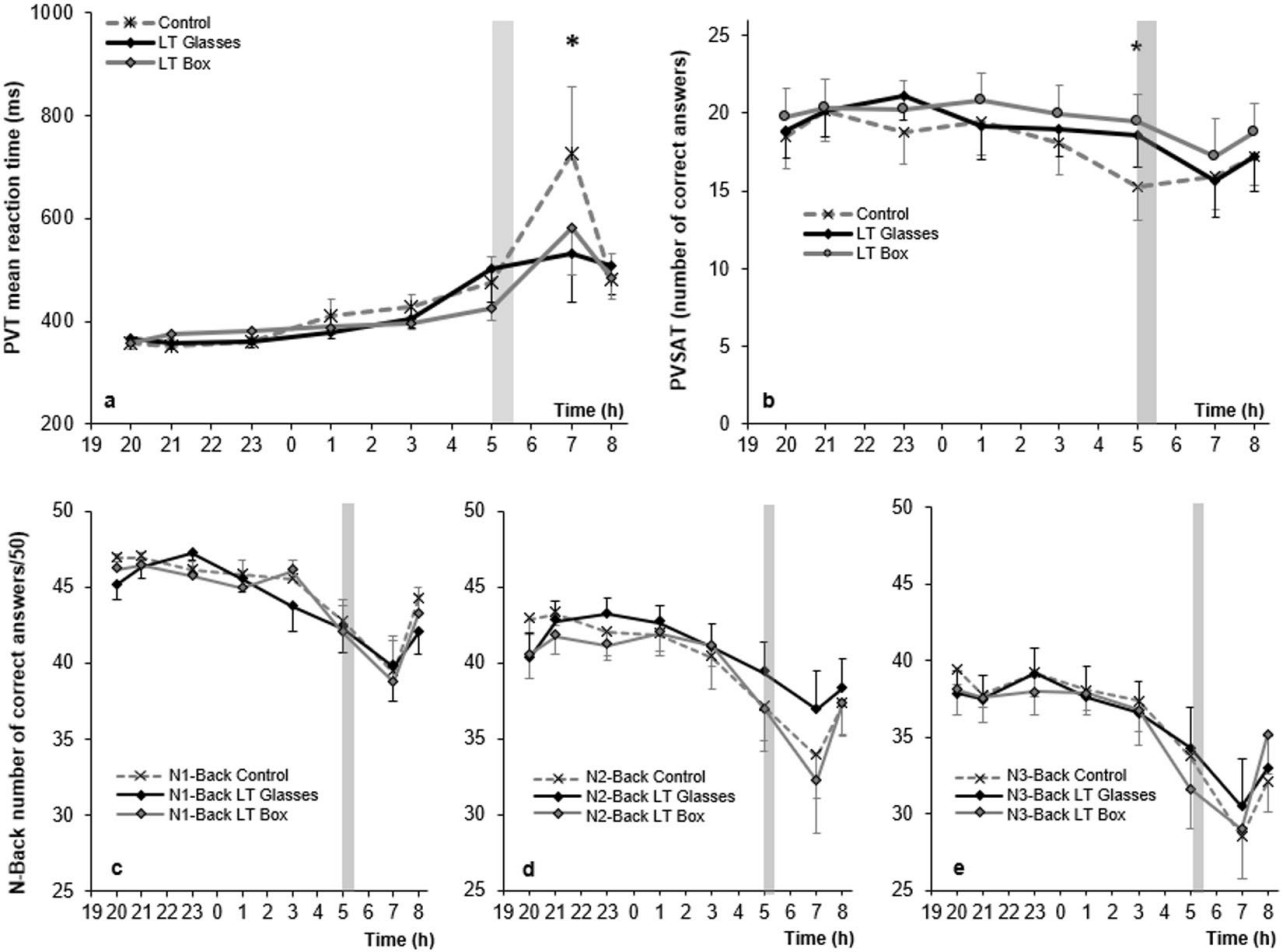
Cognition assessment by multiple computed tasks between different lighting conditions. Horizontal axes are the time (hours) of the sleep deprived night; vertical axis is for (**a**) the Psychomotor Vigilance Task (PVT) mean reaction time in ms. (**b**) the Paced Visual Serial Addition Task (PVSAT) mean number of correct answers with a maximum of 25, and (**c**–**e**) the mean number of correct answers with a maximum of 50 for respectively the Verbal 1-Back, 2-Back and 3-Back tasks. For each group, marks are the mean, bars are the SEM. Bright light therapy is administered at 5am for 30 minutes (gray rectangle). *Control vs. LT < 0.05.

Reaction time and sustained attention measured by PVT differed only at 7:00, when reaction time was significantly shorter in two LT conditions (p < 0.05) compared to control (Fig. 2a).

The number of correct responses on the PVSAT was stable and similar in all conditions until 5:00, when a drop was observed in the number of correct responses in the control condition while LT box and glasses kept a stable level of correct responses compared to control (p < 0.05 for both active LT vs. control). This difference became non-significant at the 7:00 and 8:00 time points (Fig. 2b).

**Figure 3:**
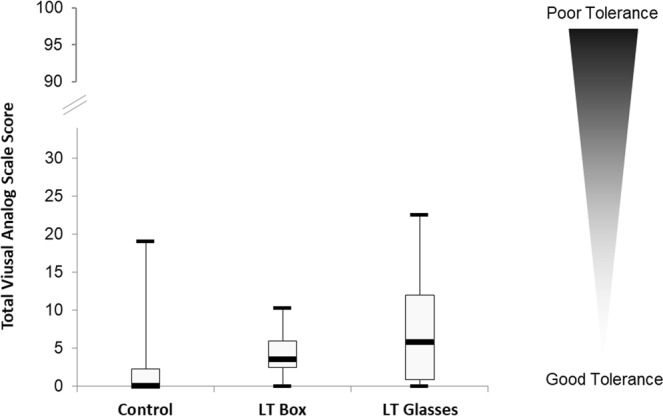
Global tolerance assessment by total V.A.S. score between different lighting conditions.Ten VAS on ten symptoms (see Methods) were administered to subjects after the light pulse. Each VAS was scored from 0 to 10, so that the maximum total VAS score was 100. Dispersion of Total VAS score is represented in a Box & Whiskers Plot design with respectively minimum, Q1, median, Q3 and maximum.

No significant differences were observed in working memory between groups as measured by the Verbal 1-back (Fig. 2c), 2-back (Fig. 2d) and 3-back (Fig. 2e) tasks. Nevertheless, there was a tendency for individuals using the LT glasses condition to respond more correctly than the other conditions at the 5:00 and 7:00 time points in the 2-back task.

### Mood

Whereas negative affects evaluated by PANAS remained stable throughout the sleep-deprivation night, positive affects decreased progressively without any statistical differences between the conditions. Of note, at the 5:00 and 7:00 time points, a slightly higher positive affect and lower negative affect were observed for both LT conditions compared to control but these trends were not statistically significant (Table 2, Supplementary Fig. S1**)**.

**Table 2:** Comparison of clinical assessments at different time points between 3 light conditions. n = 18.

Time	Control	LT Glasses	LT Box	Comparison (*p*-value)		
				Glasses vs. Control	Box vs. Control	Glasses vs. Box
	*KSS mean score*					
3am	3.84 ± 0.33	3.74 ± 0.20	3.84 ± 0.33	ns	ns	ns
5am	5.05 ± 0.41	4.05 ± 0.42	3.94 ± 0.61	<0.05	<0.05	ns
7am	6.21 ± 0.41	4.68 ± 0.53	5.42 ± 0.50	<0.05	<0.05	ns
8am	4.94 ± 0.49	4.89 ± 0.53	4.94 ± 0.49	ns	ns	ns
	*PVT mean reaction time (ms)*					
3am	427.6 ± 24.2	405.8 ± 20.1	396.2 ± 12.9	ns	ns	ns
5am	475.9 ± 50.0	501.11 ± 65.5	426.0 ± 25.0	ns	ns	ns
7am	726.6 ± 128.6	532.3 ± 94.5	581.7 ± 93.1	<0.05	<0.05	ns
8am	480.1 ± 51.6	508.6 ± 58.2	483.3 ± 41.6	ns	ns	ns
	*PVSAT number of correct responses (/25)*					
3am	18.11 ± 2.09	18.95 ± 1.79	19.95 ± 1.81	ns	ns	ns
5am	15.21 ± 2.13	18.56 ± 2.07	19.41 ± 1.83	<0.05	<0.05	ns
7am	15.89 ± 2.08	15.67 ± 2.33	17.19 ± 2.41	ns	ns	ns
8am	17.22 ± 1.87	17.16 ± 2.23	18.79 ± 1.86	ns	ns	ns
	*1-Back Task number of correct responses (/50)*					
3am	45.47 ± 0.63	43.68 ± 1.62	46.05 ± 0.66			
5am	42.68 ± 1.44	42.26 ± 1.55	41.94 ± 1.79			
7am	39.37 ± 2.37	39.74 ± 2.30	38.68 ± 2.80			
8am	44.16 ± 0.78	41.95 ± 1.40	43.16 ± 1.27			
	*2-Back Task number of correct responses (/50)*					
3am	40.35 ± 2.05	41.00 ± 1.60	41.06 ± 1.33			
5am	37.06 ± 2.22	39.35 ± 1.97	37.00 ± 2.86			
7am	33.82 ± 2.77	36.88 ± 2.62	32.18 ± 3.42			
8am	37.24 ± 2.00	38.29 ± 1.92	37.29 ± 2.14			
	*3-Back Task number of correct responses (/50)*					
3am	37.38 ± 1.79	36.56 ± 2.08	36.75 ± 2.30			
5am	33.75 ± 1.98	34.31 ± 2.66	31.60 ± 2.57			
7am	28.5 ± 3.38	30.47 ± 3.06	29.00 ± 3.22			
8am	32.06 ± 2.12	32.93 ± 2.10	35.13 ± 2.53			
	*PANAS Negative Affects (/5)*					
3am	1.28 ± 0.12	1.29 ± 0.12	1.30 ± 0.11			
5am	1.37 ± 0.12	1.28 ± 0.10	1.32 ± 0.10			
7am	1.39 ± 0.12	1.25 ± 0.09	1.29 ± 0.10			
8am	1.38 ± 0.14	1.29 ± 0.12	1.34 ± 0.15			
	*PANAS Positive Affects (/5)*					
3am	2.24 ± 0.20	2.15 ± 0.17	2.26 ± 0.15			
5am	2.07 ± 0.18	2.23 ± 0.21	2.21 ± 0.20			
7am	1.78 ± 0.17	1.99 ± 0.17	1.87 ± 0.16			
8am	2.01 ± 0.19	1.84 ± 0.12	1.99 ± 0.16			

Legend: Values are mean ± SEM, or N (%).

LT = light therapy; KSS = Karolinska Sleepiness Scale; PVT = Psychomotor Vigilance Task; PVSAT = Paced Visual Serial Attention Task; PANAS = Positive and Negative Affect Schedule.

### Light therapy tolerance

All three conditions were well tolerated with mean scores under 3/10 for all assessed side effects from a ten-point visual analog scale (VAS). Figure 3 illustrates a comparison of the tolerance total score between different light conditions (control, box, or glasses).

No statistical differences were observed between LT glasses and box regarding the VAS total score and sub-scores for headache, nausea, dizziness, visual blur, visual discomfort, abdominal pain, palpitation, dry eyes, agitation, anxiety, and irritability (Table 3). Compared to control, glasses were associated with more visual discomfort (p = 0.006), headache (p = 0.02) and a poorer total VAS score (p = 0.001). Boxes also had a significantly poorer total VAS score compared to control (p = 0.003).

**Table 3:** Tolerance assessment between different light conditions (control, box, or glasses).

Symptom	VAS	Control	LT Glasses	LT Box	Difference (*p*-value)		
					Control vs. Glasses	Controlvs. Box	LT Glasses vs. Box
Visual blur	/10	0.81 ± 0.41	1.88 ± 0.56	1.09 ± 0.39	0.096^†^	0.544	0.258
Visual discomfort	/10	0.80 ± 0.50	2.97 ± 0.72	1.16 ± 0.27	**0**.**006**	0.053**†**	0.103
Dry eyes	/10	0.46 ± 0.31	0.87 ± 0.64	0.98 ± 0.54	0.773	0.435	0.644
Dizziness	/10	0.06 ± 0.06	0.53 ± 0.31	0.11 ± 0.09	0.418	0.795	0.544
Headache	/10	0.02 ± 0.02	1.14 ± 0.41	0.34 ± 0.21	**0**.**018**	0.563	0.103
Abdominal pain	/10	0	0.25 ± 0.16	0.24 ± 0.24	0.418	0.795	0.624
Agitation	/10	0	0.11 ± 0.08	0.04 ± 0.04	0.583	0.795	0.795
Irritability	/10	0	0.86 ± 0.51	0	0.172		0.172
Nausea	/10	0	0	0			
Palpitation	/10	0	0	0			
Anxiety	/10	0	0	0			
TOTAL	/100	2.15 ± 1.08	8.61 ± 2.01	3.96 ± 0.60	**0**.**001**	**0**.**003**	0.163

VAS = Visual Analog Scale; LT = Light Therapy. Values are mean ± SEM.

Bold = *p*-value < 0.05; ^†^*p*-value < 0.10.

VAS were then separated into 3 larger categories: mild (VAS-score from 0 to 3.3/10), moderate (from 3.4 to 6.6/10) or severe (from 6.7 to 10/10). Only 5 severe VAS were recorded among the 540 VAS filled by the subjects in the study. In the control condition, one subject complained about severe visual discomfort; in the LT box condition, one subject complained about severe dry eyes; in the LT glasses condition, two subjects complained about severe visual discomfort and 1 about severe irritability (Supplementary Fig. S2).

## Discussion

This study has several key findings and first confirmed that one night of sleep deprivation induced a progressive alteration of alertness, cognition, and mood. In these sleep-deprived individuals we showed that LT can significantly enhance vigilance (with greater prolonged effect of LT glasses compared to LT boxes two hours after light exposure), cognition and also tends to improve mood. No differences between LT devices, either used as a box or glasses, were demonstrated. Such LT effects on alertness and cognition altered by sleep loss may serve to decrease the risk of accidents for night workers when a 30-minute bright light intervention is administered at 5 am. Another interesting finding is the well-tolerated and good safety profiles of both LT devices in these individuals that are sleep deprived and very sensitive to discomforts. Of note, these LT were administered after a night with very low dim-light at 8 lux and were exposed without transition to bright LT, which may have increase side effects.

Bright light administration as well as protection against natural light and administration of melatonin have previously been used as strategies to effectively help night shift workers to adapt to their schedules^18^. However, these studies aimed at altering the subjects’ circadian phase, which may not be appropriate, especially in those on a rapidly rotating shift schedule or who work only occasionally at night, for whom a circadian rhythm delay or advance may increase their difficulties and sleep loss. Few studies investigated the direct alerting and performance-enhancing effects of light to date, and have found that light administered in the early night as well as throughout the night successfully improves alertness and cognition in subjects who are sleep deprived at night^19–22^. Weisgerber *et al*. found that 45 minutes of light applied in the morning after six hours of sleep deprivation (starting at the subject’s usual bed time) had positive effects on simulated driving following light exposure, but not on reaction time as measured by the PVT, while sleepiness measured by KSS decreased slightly but only after the 44 minute simulation^23^.

In this context, our main objective has been to provide further insight on the potential risks and benefits of early morning bright light in sleep deprived subjects. This could prove to be an effective therapy to augment productivity and safety for those who work an occasional night shift but do not show an interest in phase delaying their circadian rhythm as they must be able to rapidly re-adapt to a daytime schedule. The timing of our 5 am LT was selected based on the timing of typical night shifts, as unlike the morning LT timing by Weisgerber *et al*., most work schedules are not adapted to the individual’s circadian rhythm. We then measured parameters of alertness, cognition (including reaction time), and mood throughout the night and into the morning in order to chart the evolution of these parameters as the previous study left results that were somewhat unclear regarding reaction time and sleepiness.

Interestingly, our results indicate that alertness, cognition and mood decrease with sleep deprivation throughout the night, probably as a result of the build-up of homeostatic sleep pressure combined with influences of the circadian phase influence. The improvement in all parameters at 8:00 seems to be a result of the circadian drive overriding the homeostatic process, as previously been reported^24,25^. Sleepiness as well as negative effects on reaction time and sustained attention can be curbed with a 30-minute LT applied at 5:00 with both an immediate and a prolonged two-hour effect before returning to control levels. Mood was not significantly improved by LT. Nevertheless, a tendency to better mood in the LT groups after LT exposure was observed, which is in line with earlier results^26^. These effects are deemed to be a result of light’s non-circadian influence counteracting the sleep homeostat through direct stimulation of alertness-promoting neural systems^12,27^. PANAS has already been evaluated in condition of sleep deprivation, and our results are consistent with a previous study^28^. Finally, our results, assessing a single session of LT on mood after sleep deprivation, should not be compared to the well-demonstrated beneficial effects of LT during several days on depression^29^. To observe such antidepressant effects, LT needs to be administered for weeks.

Our secondary goal was to determine whether light administration with a portable LT device is as efficient as a traditional LT box. Three studies have reported beneficial effects of LT glasses on parameters such as well-being at work in an environment without natural light^30^, sleep and mood in adolescents^31^, and melatonin suppression in healthy subjects^32^. However, these studies did not compare the effects of LT glasses with traditional light boxes. In our study, we have found that these two devices have comparable effects on sleepiness and sustained attention.

Nevertheless, the two devices emit light at different wavelength spectrums and intensity, which can have different physiological consequences. While the conventional LT box used in this study (EnergyLight®) emits a polychromatic white light at 10,000 Lux, the LT glasses used in this study (Luminette®) emits a blue-enriched polychromatic white light, which may exert stronger effects on physiology and behavior. Indeed, the non-image forming effects of light are primarily mediated by melanopsin-based phototransduction with a peak sensitivity of melanopsin located within the blue spectrum^33,34^. The similarity of the results between LT glasses and box suggests however that polychromatic white light without blue enrichment is sufficient to produce beneficial effects, as previously observed in studies examining melatonin suppression by light^35^. Sasseville *et al*. also recently demonstrated that LT at the middle of the night (3 am) during 30 minutes at 500 µW/cm² could be as effective with or without the contribution of short-wavelength on subjective alertness and energy, thus suggesting that direct non-image forming effects of light are also mediated by rods and cones^36^. Our results are consistent with those of Sasseville *et al*., as we didn’t show differences in our study between white polychromatic LT (Philips) and blue-enriched LT (Lucimed): thus, bright light therapy produces its effect non solely via melanopsin pathways. However, two hours after LT, vigilance improvement induced by LT glasses was higher than that produced by LT boxes, suggesting a more prolonged effect of LT glasses which might result from greater blue light enrichment. Future studies with different monochromatic lights will help us to disentangle the different pathways of the non-image forming effects of light.

Tolerance analysis of LT boxes and LT glasses resulted in statistical differences regarding global discomfort compared to control, especially concerning headaches, visual blur and visual discomfort, which were generally higher for LT glasses. Nevertheless, the overall discomfort remained quite low with a total VAS score < 10/100 for all conditions, and all sub-scores < 3/10 indicating a high global well tolerance of both boxes and glasses. Of note, the light therapy in this study was administered after a complete night of sleep deprivation in condition of a very low dim light < 8 Lux, without any transition phase. This could have led to a poorer global tolerance for both LT glasses and box. Indeed, previous studies reported the effects of sleep loss on emotions^37^. Thus, the tolerance of LT glasses and box may be even higher in individuals who are not sleep deprived.

The current study includes some limitations. We should first acknowledge that our study did not allow for a full assessment of the risks of this intervention, notably, the long-term effects on the circadian system through possible phase advancement. We have not evaluated the circadian consequences of the 30-minute LT. Also, ambient light was dim light < 8 Lux aiming to avoid any melatonin suppression by light excepted during bright light therapy at 5 am, and so all participants were in a controlled condition during the night at lab. Thus this is not an ecological design but rather a proof of concept design assessing direct effects of short duration LT after sleep deprivation.

Future studies should measure for instance the circadian rhythms of melatonin secretion and core body temperature, to be able to weight the circadian effect against the observed benefits of direct early morning LT, and consequently recommend this light therapy strategy.

In conclusion, we have found some promising results regarding the positive, direct and short-term effects of light on vigilance and cognition, all of which can have important practical applications for night shift workers.

## Methods

### Population

Volunteers were recruited by advertisements at the Sleep Disorders Center & International Centre for Research in ChronoSomnology (CIRCSom), at the University Hospital of Strasbourg, France. Inclusion criteria were: age of 18–35 years; possession of medical insurance; no major health problems; no known sleep disturbances or disorders; and for women, use of hormonal contraceptives and no pregnancy or breastfeeding. The non-inclusion criteria were: participation to another clinical trial in the previous three months; any type of shift works during the preceding year of the inclusion; all trans-meridian travels in the preceding month; failure to pass a general medical and ophthalmological evaluation; and a pathological score on questionnaires concerning quality of life (MOS SF 36^38^, score ≤ 50/100), sleep (PSQI^39^, score ≥ 11/21, and ISI^40^, score > 15/28), vigilance (ESS^41^, score ≥ 11/24), chronotype (H&O^42^, score < 30 or > 70/83) and mood (BDI-II^43^, score > 8/63). Subjects were asked to respect regular sleep-wake rhythms starting a week before the beginning of the study until the end of the study, controlled by a sleep log. Subjects were asked not to consume alcohol or coffee during the day preceding each night spent in the laboratory.

### Ethics

This research project was carried out in accordance with the guidelines for clinical research as stated in the Helsinki Declaration, and was approved by the local ethical committee (*Comité de Protection des Personnes*, CPP Est) and the French National Public Health Agency (*Agence Nationale de Sécurité du Médicament et des produits de santé*, ANSM, research protocol number 2014-A01529-38). Data recorded in this study were protected accordingly to the statements of the national committee (*Commission Nationale de l’Informatique et des Libertés*, CNIL). Written informed consent was obtained from all the participants.

### Study design

Subjects participated to three sleep deprivations, with a recovery period of 7 to 28 days between each sleep deprivation in the study. The 24 hour sleep deprivation began first at home from 8 am to 8 pm while subjects were asked not to sleep nor to lie down, then from 8 pm to 8 am at the lab CIRCSom while subjects staying in the lab, in a controlled ambient light < 8 Lux (verified by lightmeter Testo® 540). During the in-lab nights, subjects’ activities were free (for example: reading, drawing, …) excepted any physical activities and activities with electronical devices or devices emitting light. Subjects were monitored by the investigator to be sure they did not fall asleep. Snacking were hourly proposed to the subjects, without mention of time, in order to avoid time cues. After each of the three sleep deprivations spent at the CIRCSom, each subject was exposed to three different lighting conditions – in an order determined by computer randomization – in this cross-over design study: (1) box: EnergyLight® LT by Philips, (2) glasses: Luminette® LT by Lucimed, and (3) control condition: 8 lux control. Throughout each of these three separate nights, which lasted from 8 pm to 8am the next morning, subjects were asked to respond to a battery of neuropsychological tests, chosen for their repeatability over time, assessing vigilance (Karolinska Sleepiness Scale – KSS^44^, sustained attention (Psychomotor Vigilance Task – PVT^45^; Paced Visual Serial Addition Task – PVSAT^46^, working memory (Verbal 1, 2 & 3-back Tasks^47^, and mood (Positive and Negative Affect Schedule – PANAS^48^ at eight time points (Supplementary Fig. S3). After each sleep deprivation night, subjects were given time to nap, and benefited from a medical examination before being allowed to leave the hospital.

### Light therapy

Subjects were exposed to LT starting at 5 am for 30 minutes during the three trials using box, glasses, or light control, depending on the randomization.

The control consisting of a non-active white light emitted from conventional incandescent light bulbs with a filter limiting the luminosity to a maximum of 8 lux. The intensity of ambient lighting, including light emitted from the computer screens used for the test battery, was controlled to be less than 8 Lux, as determined by the lightmeter Testo® 540. The control light during light therapy was the same as the ambient light.

The EnergyLight® (CE marking 86443CE02) is a medical device for LT and emits white light (from compact fluorescent lamps) with an adjustable illuminance of up to 10,000 Lux and color temperature of 4,000 °K to be placed at a distance of 15–50 cm from the eyes. For this study, the lamp was set up to 10,000 Lux and at a distance of 50 cm.

The Luminette® (CE marking BE07/71114) is a pair of glasses equipped with LT and developed more recently^16,30,49^. The glasses contain diodes emitting a blue-enriched white light reflecting to the retina at 2,000 Lux via a holographic system in order to ensure correct penetration into the eye without impeding vision.

Subjects were asked to rate their tolerance to each of the three LT conditions 30 minutes after the exposure using ten-point visual analog scales (VAS) which assessed ten possible side-effects: headache, dizziness, blurred vision, dry eyes, visual discomfort, anxiety, irritability, agitation, nausea, and palpitation. Scores ranged from “no symptoms” (score 0) to “worst imaginable experience of the symptom” (score 10). In addition, for each LT condition, these VAS scores were added resulting in a “Total VAS Score” ranging from 0 to 100 points.

### Statistics

A mixed-model analysis of variance for repeated measures (PROC MIXED) was used for all analyses (package SAS version 9.1; SAS Institute, Cary, NC, USA). For each variable studied in the test battery (subjective vigilance level as determined by the KSS, subjective mood as determined by the PANAS, and cognitive performance as determined by the PVT, the PVSAT and the N-Back), differences were compared between lighting conditions (control, box, or glasses) and time point during the night, as well as two random factors “subject” and “order of the session”. Contrasts were assessed with the LSMEANS statement and p values were based on Kenward-Roger’s corrected degrees of freedom^50^. Results are expressed as mean ± SEM. From a previous study with similar design, we estimated that a sample size of 18 subjects would allow to detect differences with a statistical power of 0.80 and a significance level at 0.05^17^.

Tolerance was evaluated by comparing between each condition the mean score obtained from the ten-point scores from VAS. We compared the individual VAS scores for headache, nausea, dizziness, visual blur, visual discomfort, abdominal pain, palpitation, dry eyes, agitation, anxiety, and irritability, and the total VAS score. Non-parametric tests were performed (Mann-Whitney U test) due to the non-gaussian distributions.

## Supplementary information

Supplementary Information

## Data Availability

The datasets generated during and/or analyzed during the current study are available from the corresponding author on reasonable request.

## References

[1] Killgore WD (2010). Effects of sleep deprivation on cognition. Prog. Brain. Res..

[2] Raven F, Van der Zee EA, Meerlo P, Havekes R (2018). The role of sleep in regulating structural plasticity and synaptic strength: implications for memory and cognitive function. Sleep. Med. Rev..

[3] Wilkinson RT, Edwards RS, Haines E (2013). Performance following a night of reduced sleep. Psychon. Sci..

[4] Forest G, Godbout R (2000). Effects of sleep deprivation on performance and EEG spectral analysis in young adults. Brain Cogn..

[5] Dinges DF (1997). Cumulative sleepiness, mood disturbance, and psychomotor vigilance performance decrements during a week of sleep restricted to 4-5 hours per night. Sleep..

[6] Brum MCB, Filho FFD, Schnorr CC, Bottega GB, Rodrigues TC (2015). Shift work and its association with metabolic disorders. Diabetol. Metab. Syndr..

[7] Kervezee L, Kosmadopoulos A, Boivin DB (2018). Metabolic and cardiovascular consequences of shift work: the role of circadian disruption and sleep disturbances. Eur. J. Neurosci..

[8] Kang MY, Kwon HJ, Choi KH, Kang CW, Kim H (2017). The relationship between shift work and mental health among electronics workers in South Korea: a cross-sectional study. PLoS ONE..

[9] Kecklund G, Axelsson J (2016). Health consequences of shift work and insufficient sleep. BMJ..

[10] Fischer D, Lombardi DA, Folkard S, Willetts J, Christiani DC (2017). Updating the “Risk Index”: a systematic review and meta-analysis of occupational injuries and work schedule characteristics. Chronobiol. Int..

[11] Cajochen C (2007). Alerting effects of light. Sleep Med. Rev..

[12] Hubbard J, Ruppert E, Gropp CM, Bourgin P (2013). Non-circadian direct effects of light on sleep and alertness: lessons from transgenic mouse models. Sleep Med. Rev..

[13] Leger D, Duforez F, Gronfier C (2018). & le Groupe consensus chronobiologie et sommeil de la Société française de recherche et médecine du sommeil (SFRMS). [Treating circadian sleep-wake disorders by light]. Presse Med..

[14] Even C, Schröder CM, Friedman S, Rouillon F (2008). Efficacy of light therapy in nonseasonal depression: a systematic review. J. Affect. Disord..

[15] Maruani, J. & Geoffroy, P. A. Bright light as a personalized precision treatment of mood disorders. *Front*. *Psychiatry*. **10**, https://www.frontiersin.org/articles/10.3389/fpsyt.2019.00085/full (2019). 10.3389/fpsyt.2019.00085 PMC6405415 30881318

[16] Geoffroy PA (2018). Bright light therapy in the morning or at mid-day in the treatment of non-seasonal bipolar depressive episodes (LuBi): study protocol for a dose research phase I/II trial. Psychiatry Investig..

[17] Viola AU (2007). PER3 polymorphism predicts sleep structure and waking performance. Curr. Biol..

[18] Burgess HJ, Sharkey KM, Eastman CI (2002). Bright light, dark and melatonin can promote circadian adaptation in night shift workers. Sleep Med. Rev..

[19] Badia P, Myers B, Boecker M, Culpepper J, Harsh JR (1991). Bright light effects on body temperature, alertness, EEG and behavior. Physiol. Behav..

[20] Campbell SS, Dawson D (1990). Enhancement of nighttime alertness and performance with bright ambient light. Physiol. Behav..

[21] Rüger M, Gordijn MCM, Beersma DGM, de Vries B, Daan S (2006). Time-of-day-dependent effects of bright light exposure on human psychophysiology: comparison of daytime and nighttime exposure. Am. J. Physiol. Regul. Integr. Comp. Physiol..

[22] Wright KP, Badia P, Myers BL, Plenzler SC (1997). Combination of bright light and caffeine as a countermeasure for impaired alertness and performance during extended sleep deprivation. J. Sleep Res..

[23] Weisgerber DM, Nikol M, Mistlberger RE (2017). Driving home from the night shift: a bright light intervention study. Sleep Med..

[24] Carrier J, Monk TH (2000). Circadian rhythms of performance: new trends. Chronobiol. Int..

[25] Cajochen C, Blatter K, Wallach D (2004). Circadian and sleep-wake dependent impact on neurobehavioral function. Psychol. Belg..

[26] Pilcher JJ, Huffcutt AI (1996). Effects of sleep deprivation on performance: a meta-analysis. Sleep..

[27] Tsai JW (2009). Melanopsin as a sleep modulator: circadian gating of the direct effects of light on sleep and altered sleep homeostasis in Opn4(−/−) mice. PLoS Biol..

[28] Franzen PL, Siegle GJ, Buysse DJ (2008). Relationships between affect, vigilance, and sleepiness following sleep deprivation. J. Sleep Res..

[29] Terman M, Terman JS (2005). Light therapy for seasonal and nonseasonal depression: efficacy, protocol, safety, and side effects. CNS Spectr..

[30] Bragard I, Coucke PA (2013). Impact of the use of Luminette® on well-being at work in a radiotherapy department. Cancer Radiother..

[31] Bonnar D (2015). Evaluation of novel school-based interventions for adolescent sleep problems: does parental involvement and bright light improve outcomes?. Sleep Health..

[32] Claustrat B (2010). Suppression of melatonin secretion in healthy subjects with eyeglass LED delivery system. Neuro. Endocrinol. Lett..

[33] Hughes S, Hankins MW, Foster RG, Peirson SN (2012). Melanopsin phototransduction: slowly emerging from the dark. Prog. Brain Res..

[34] van der Meijden WP (2015). Post-illumination pupil response after blue light: reliability of optimized melanopsin-based phototransduction assessment. Exp. Eye Res..

[35] Revell VL, Skene DJ (2007). Light-induced melatonin suppression in humans with polychromatic and monochromatic light. Chronobiol. Int..

[36] Sasseville A, Martin JS, Houle J, Hebert M (2015). Investigating the contribution of short wavelengths in the alerting effect of bright light. Physiol & Behav..

[37] Goldstein AN, Walker MP (2014). The role of sleep in emotional brain function. Annu. Rev. Clin. Psychol..

[38] Ware JE, Sherbourne CD (1992). The MOS 36-item short-form health survey (SF-36). I. Conceptual framework and item selection. Med. Care..

[39] Buysse DJ, Reynolds CF, Monk TH, Berman SR, Kupfer DJ (1989). The Pittsburgh sleep quality index: a new instrument for psychiatric practice and research. Psychiatry Res..

[40] Bastien CH, Vallières A, Morin CM (2001). Validation of the Insomnia Severity Index as an outcome measure for insomnia research. Sleep Med..

[41] Johns MW (1991). A new method for measuring daytime sleepiness: the Epworth sleepiness scale. Sleep..

[42] Taillard J, Philip P, Chastang JF, Bioulac B (2004). Validation of Horne and Ostberg morningness-eveningness questionnaire in a middle-aged population of french workers. J. Biol. Rhythms..

[43] Beck AT, Steer RA, Ball R, Ranieri W (1996). Comparison of Beck Depression Inventories -IA and -II in psychiatric outpatients. J. Pers. Assess..

[44] Akerstedt T, Gillberg M (1990). Subjective and objective sleepiness in the active individual. Int. J. Neurosci..

[45] Dinges DF, Powell JW (1985). Microcomputer analyses of performance on a portable, simple visual RT task during sustained operations. Behavior Research Methods, Instruments, & Computers..

[46] Fos LA, Greve KW, South MB, Mathias C, Benefield H (2000). Paced Visual Serial Addition Test: an alternative measure of information processing speed. Appl. Neuropsychol..

[47] Kirchner WK (1958). Age differences in short-term retention of rapidly changing information. J. Exp. Psychol..

[48] Watson D, Clark LA, Tellegen A (1988). Development and validation of brief measures of positive and negative affect: the PANAS scales. J. Pers. Soc. Psychol..

[49] Holtmann M (2018). Adolescent depression: study protocol for a randomized, controlled, double-blind multicenter parallel group trial of bright light therapy in a naturalistic inpatient setting (DeLight). Trials..

[50] Kenward MG, Roger JH (1997). Small sample inference for fixed effects from restricted maximum likelihood. Biometrics..

