# Supplementary material for: Light therapy with boxes or glasses to counteract effects of acute sleep deprivation
Source: Sci Rep. 2019 Dec 2;9:18073. doi: 10.1038/s41598-019-54311-x (PMC6889287; doi:10.1038/s41598-019-54311-x)
Supplement: Supplementary file 1 — Supplementary Information [file 41598_2019_54311_MOESM1_ESM.docx]

**Full title:**

**Light therapy with boxes or glasses to counteract effects of acute sleep deprivation**

Comtet, Henri^1,2*^, Geoffroy, Pierre A^1,2,3^; Kobayashi Frisk, Mio^1,2^; Hubbard, Jeffrey^1,2^; Robin-Choteau, Ludivine^1,2,4^; Calvel, Laurent^2,5^; Hugueny, Laurence^1,2^; Viola, Antoine U^1,2^; Bourgin, Patrice^1,2,5*^.

1. Sleep Disorders Center & CIRCSom (International Research Center for ChronoSomnology), University Hospital, 1 place de l’Hôpital, 67000 Strasbourg, France;
2. CNRS UPR 3212, Institute for Cellular and Integrative Neurosciences, 8 rue du Général Rouvillois, 67000 Strasbourg France;
3. Paris Diderot University - Paris VII, 5 Rue Thomas Mann, 75013 Paris, France;
4. CEED (European Center for Diabetes Studies), Boulevard Leriche, 67200 Strasbourg, France;
5. Fédération de Médecine Translationnelle de Strasbourg (FMTS), Strasbourg University, 4 rue Kirschleger, 67085 Strasbourg, France.

***Corresponding Author:**

Dr Comtet Henri

International Research Center for ChronoSomnology

University Hospital,

1 place de l’hôpital, 67000 Strasbourg

[henri.comtet@chru-strasbourg.fr](mailto:henri.comtet@chru-strasbourg.fr)

**Supplementary Information**

**Figure S1**


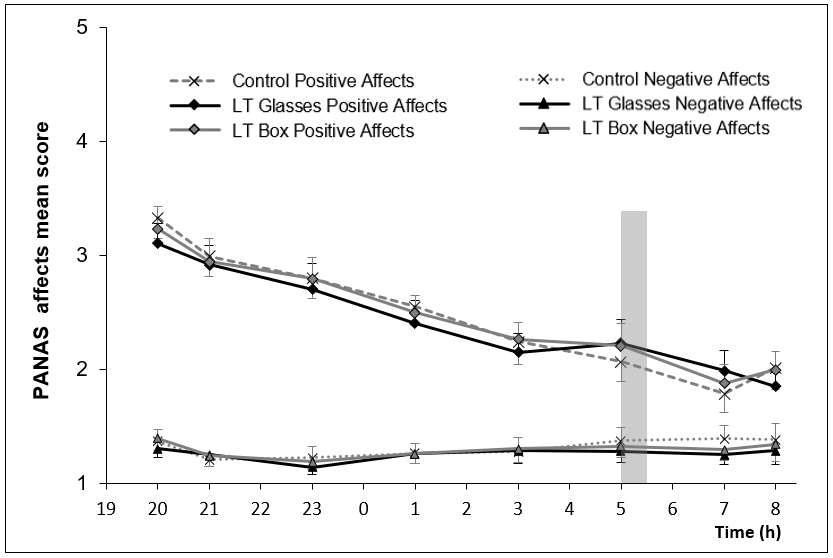


***Figure S1: Mood evaluated by Positive And Negative Affect Schedule (PANAS).***

*Horizontal axis is the time (in hours) of the sleep deprived night; vertical axis is the PANAS mean score. For each group (Control = broken line; LT Glasses = black line; LT Box = gray line), marks are the mean, bars are the SEM. PANAS is scored from 1 to 5. Bright light therapy is administered at 5am for 30 minutes (gray rectangle).*

**Figure S2**


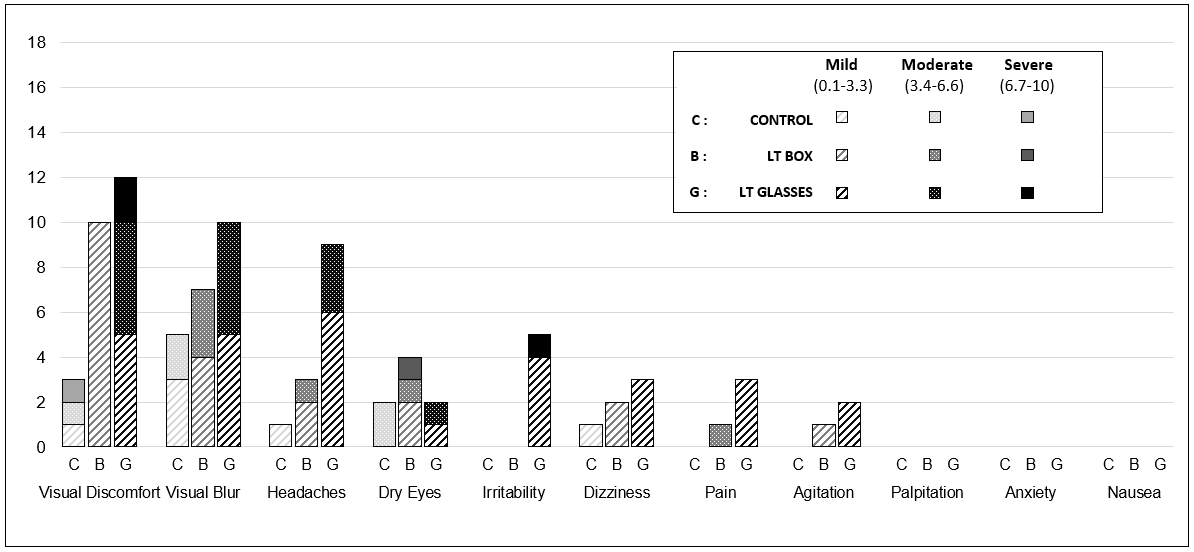


***Figure S2: Number of subjects with a tolerance complaint.*** *Ten VAS on ten symptoms were administered to subjects after the light pulse. Each VAS was scored from 0 to 10 and each result was separated in three categories: Mild (VAS from 0.1 to 3.3); Moderate (VAS from 3.4 to 6.6); Severe (VAS from 6.7 to 10). The figure represents the number of subjects complaining about each symptom, separated by intensity and by lighting condition. Control condition in low gray, LT Box in medium gray, LT Glasses in black. Mild intensity in stripes, Moderate intensity in points, Severe intensity in plain color.*

**Figure S3**


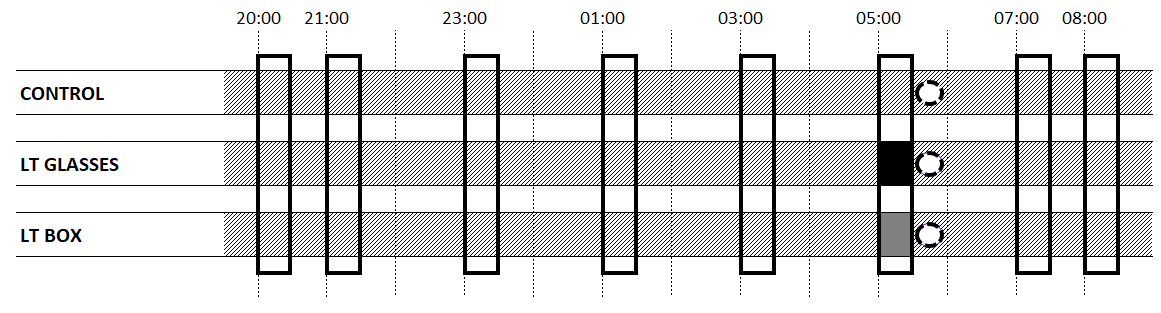


**Figure S3: Study Design**

Subjects were place in a controlled ambient dim light < 8 Lux from 8 pm to 8 am the following day (gray dashed background). All subjects took part in all three sleep deprivation night, in a random order (cross-over study). They performed repeated evaluation (black frames) throughout the night, and were exposed to light therapy (LT) at 5 am for 30 minutes (Control = dim light, LT Glasses = black background, LT Box = grey background). Tolerance evaluation of the LT was performed for each condition just after the light exposure (circles).
